# Potential for intranasal drug delivery to alter cerebrospinal fluid outflow via the nasal turbinate lymphatics

**DOI:** 10.1186/2045-8118-11-4

**Published:** 2014-02-15

**Authors:** Harold Kim, Sara A Moore, Miles G Johnston

**Affiliations:** 1 Department of Laboratory Medicine and Pathobiology, University of Toronto and Sunnybrook Research Institute, Toronto, Canada

## Abstract

**Background:**

Cerebrospinal fluid absorption (CSF) at the cribriform plate is mediated by direct extracranial connections to the lymphatic system. Given the accessibility of these pharmacologically responsive vessels we hypothesized that the rate of CSF outflow can be modulated via the intranasal delivery of drugs known to affect lymphatic contractile activity.

**Findings:**

Fluid was infused into the lateral ventricle of anesthetized sheep and inflow rate and CSF pressure measured during intranasal administration of pharmacological agents. CSF absorption was calculated at steady-state CSF pressures. The ability of a pharmacological agent to alter CSF absorption was related to the steady-state intracranial pressure (ICP), the concentration and the class of pharmacological agent delivered. An increase in drug concentration correlated with an increase in CSF absorption at high ICP (45 cm H_2_O, r = 0.42, *p* = 0.0058). Specifically, the delivery of NG-monomethyl L-arginine (L-NMMA) significantly increased CSF absorption by 2.29 fold over no treatment (2.29 ± 0.34 mL/min), while the thromboxane A2 analogue U46619 resulted in a 2.44 fold increase in CSF absorption over no treatment (2.44 ± 0.55 mL/min). Saline delivery did not significantly increase CSF absorption (0.88 ± 0.097 mL/min). A trend of increased CSF absorption upon noradrenaline delivery was observed: however, this did not reach statistical significance. Increasing drug concentrations inversely correlated with CSF outflow resistance across all drug classes (r = -0.26, *p* = 0.046).

**Conclusions:**

The administration of nebulized pharmacological agents intranasally has the potential to provide an alternate method to non-invasively modulate CSF absorption and outflow resistance.

## Introduction

The regulation of intracranial homeostasis is strictly controlled due to the finite space offered by the fixed volume of the skull. As defined by the Monroe-Kellie hypothesis, tight volumetric regulation of the cerebrospinal fluid (CSF), blood and tissue spaces is critical in maintaining fluid balance; deflections in any of these volumes outside of the autoregulatory capacity of the brain can result in cerebral edema, high intracranial pressures, and compromised cerebral perfusion. The primary mechanism to control intracranial pressure (ICP) is via regulation of CSF production and absorption within the ventricular and subarachnoid spaces [1]. Strong evidence supports a mechanism where the absorption of CSF occurs extracranially at the cribriform plate via the ethmoid turbinate lymphatics [2-4]. Indeed, a significant portion of CSF absorption occurs through the foramina and directly into the extracranial lymphatics. Restriction of this pathway results in increased resistance, decreased outflow and raised ICP [5-7].

Given the direct connection between the CSF compartment and the pharmacologically responsive lymphatic vasculature within the turbinate region, the potential exists to modulate lymphatic pumping and thus CSF absorption. Potential drugs that have been found to modulate lymphatic contractile activity include noradrenaline (NA). Indeed, NA binding at <10^-6^ M to α-adrenergic receptors can increase phasic contractions and increase flow on quiescent vessels, however NA stimulation of vessels that display spontaneous rhythmic activity results in reduced contraction force and outflow [8-12]. Similarly, the binding of thromboxaneA2, or its analogue U46619 to its’ receptors have been found to increase the synthesis of inositol-triphosphate (Ins(1,4,5)P_3_) and augment smooth muscle contractions in a concentration-dependent fashion; higher concentrations lead to vessel spasm and attenuate flow [13-17]. In contrast, the nitric oxide competitor NG-monomethyl L-arginine (L-NMMA) inhibits lymphatic contractions [18].

Utilizing these pharmacological agents, the aim of these experiments was to test whether the intranasal delivery of these drugs can alter CSF absorption. We have found that indeed this is the case and provide a proof-of-concept for the possibility to influence CSF absorption in a non-invasive fashion.

## Findings

### Materials and methods

#### Animals

After at least 48 hours for acclimatization, sheep (15–45 kg) were fasted 12 hours prior to surgery. All sheep experiments were acute followed by euthanasia with an IV injection of sodium pentobarbital into the catheterized cephalic vein using a dose of 1 mL/5 kg of Euthanyl Forte. Thirty-six animals were used in the study with 22 included in subsequent data analysis; 14 were excluded due to surgical and/or technical issues. Experiments were approved by the ethics committee at Sunnybrook and Women’s College Health Sciences Centre and conformed to the guidelines set by the Canadian Council on Animal Care and the Animals for Research Act of Ontario.

#### Surgical procedures

Sodium pentothal (15–25 mg/kg) was given intravenously to induce anesthesia. Sheep were set in a ventral position, intubated, ventilated and surgical anesthesia was maintained with 2-3% Isoflurane/oxygen. An 18–20 Ga catheter was placed in the cephalic vein for venous pressure measurements. An IV line provided hydration using lactated Ringers solution at 5 mL/kg/hr. The opposite cephalic vein was connected to a fluid-filled transducer to record venous pressure. Midsagittal and coronal incisions were made in the scalp to expose the coronal suture and the scalp and posterium pulled aside. A hole was drilled in the skull lateral to the sagittal suture and at approximately 10 degrees to the sagittal plane. Two 18 or 20 Ga angiocatheters were inserted through the hole into each lateral ventricle of the brain and sealed in place.

#### Constant pressure experiments

Using a modified protocol described in [7], a reservoir filled with lactated Ringers solution was connected to one lateral ventricular catheter while a fluid-filled transducer to measure ICP was connected to the contralateral ventricle by the second catheter. For pre-drug treatment a stable baseline ICP was recorded indicating inflow was equal to outflow, and then the reservoir was raised 5 cm. A steady-state pressure was achieved within 5 minutes and at this point the flow of lactated Ringers into the ventricle was recorded for the next 5 minutes via a drop counter. The reservoir was raised again 5 cm and the procedure repeated. When complete, the reservoir was lowered and ICP returned to baseline. Subsequently, one application of a 20 mL volume (10 mL per nostril) of either saline (n = 5) or drug was nebulized using a NasoNeb nebulizer (MedInvent, LLC, White Bear Lake, Mn, USA) and the above protocol was repeated. The drugs used were noradrenaline at 50 μM and 500 μM (n = 5), L-NMMA (Santa Cruz Biotechnology, Santa Cruz, USA) at 10 μM and 100 μM (n = 4) and U46619 (Santa Cruz Biotechnology) at 0.1 μM, 1 μM and 1 mM (n = 8). CSF pressure was monitored using a data acquisition system.

#### Data analysis

##### CSF absorption

Using the linear equations derived from each single experiment, we calculated theoretical CSF outflow rates at preset intracranial pressures of 25 cmH_2_O and 45 cmH_2_O. All analysis utilized raw outflow numbers derived from the linear regression equations.

##### Resistance

At steady-state pressure, ventricular inflow rate equals CSF outflow rate. From the regression analysis of steady-state ICP versus flow, the CSF outflow resistance can be calculated from the slope of the line. All analysis utilized slopes derived from the linear regression equations.

#### Statistical analysis

The data was analyzed using repeated measures ANOVA, post-hoc Dunnett’s, Students paired *t*-test and Pearson Correlation test. Results were deemed statistically significant at *p ≤ 0.05*.

## Results

### Intranasal administration of drug shifts intracranial pressure versus cerebrospinal fluid curves to the right

**Figure 1:**
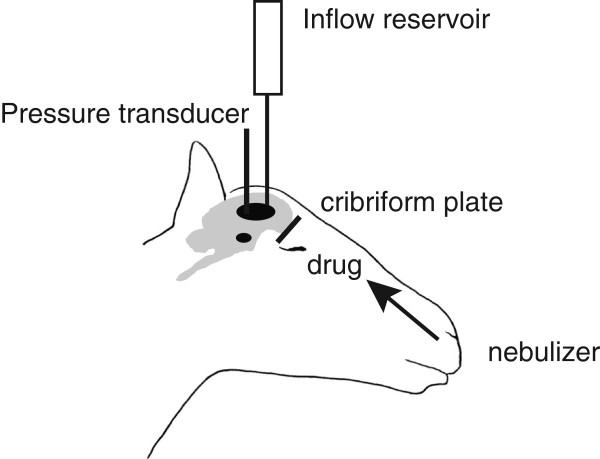
Schematic of experimental set-up.

**Figure 2:**
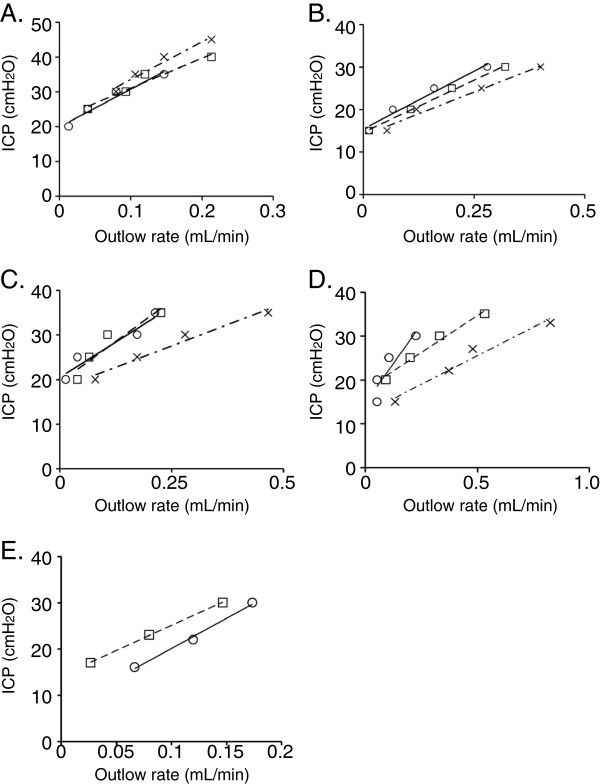
**Intracranial pressure and CSF outflow relationships upon intranasal delivery of pharmacological agents.** Shown are representative examples of pressure/flow curves of **(A)** saline delivery, **(B)** noradrenaline (NA) at 50 μM and 500 μM, **(C)** L-NMMA treated at 10 μM and 100 μM and **(D)** U46619 treated at 0.1 μM and 1.0 μM and **(E)** U46619 at 1 mM. **(A to D)** In each experiment (○) represents no treatment (NT), (□) represents the first intranasal delivery, and (×) represents the second intranasal delivery. **(E)** No treatment is represented by (○), and 1 mM U46619 represented by (□). Linear regression lines for each treatment are shown.

Given the direct connection between the CSF compartment and the extracranial lymphatics within the nasal turbinate region, we hypothesized that the nebulization of pharmacological agents into the nasal cavity may increase CSF absorption (Figure 1). While the delivery of saline (n = 5) did not alter CSF absorption, we observed that the delivery of agents such as noradrenaline (NA, n = 5), L-NMMA (n = 4) and U46619 (n = 5) at steady state intracranial pressures increased CSF absorption in a dose dependent manner relative to no drug treatment. This result also illustrated that the presence of drug lowered ICP relative to no drug treatment at a specific outflow rate (Figure 2A to E).

### CSF absorption is dependent on drug concentration

**Figure 3:**
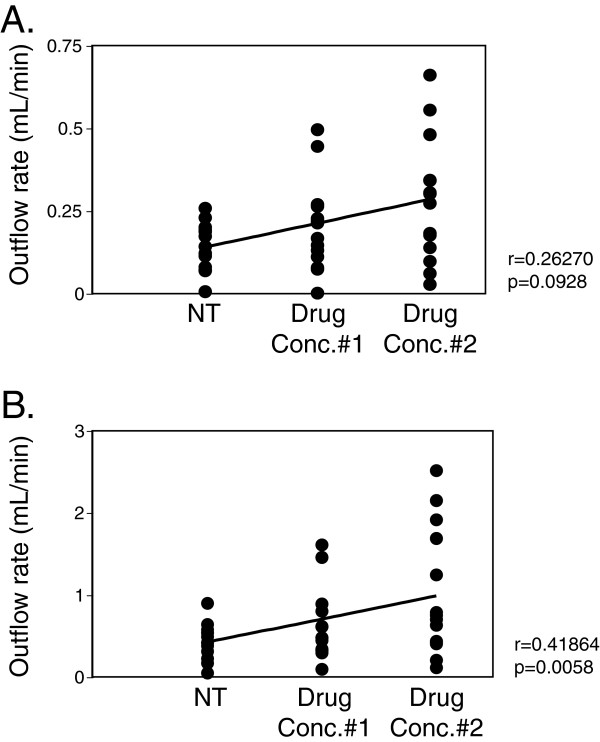
**CSF outflow correlated with drug concentration at (A) 25 cmH**_**2**_**O and (B) 45 cmH**_**2**_**O ICP.** In order to address whether a correlation existed between CSF outflow and an overall drug effect, data points from all agents tested at no drug treatment (NT), the delivery of the first set of agents (Drug Conc.#1; 50 μM NA, 10 μM L-NMMA, 0.1 μM U46619) and the delivery of the second set of agents (Drug Conc.#2; 500 μM NA, 100 μM L-NMMA, 1 μM U46619) were plotted against CSF outflow, which was calculated from linear regression analysis derived from the raw data using steady-state pressures of **(A)** 25 cmH_2_O and **(B)** by extrapolation at 45 cmH_2_O. The straight line linking the averages within each treatment group was calculated with the Pearson correlation test.

**Figure 4:**
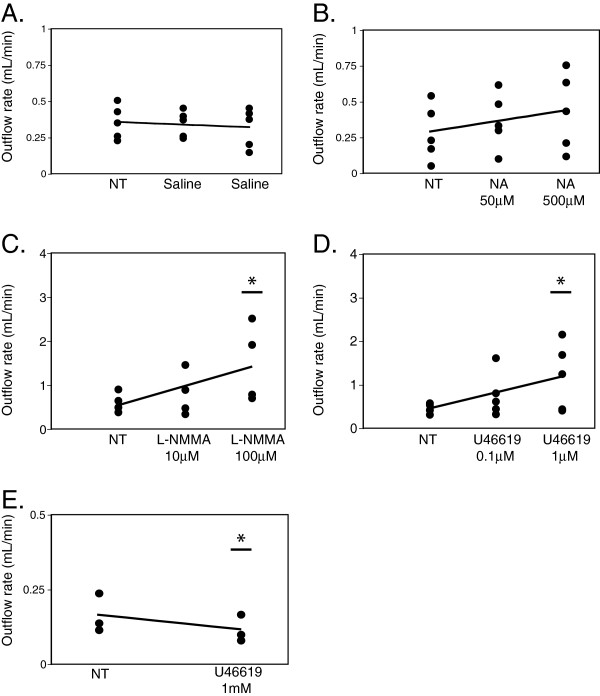
**Increasing drug dose resulted in a significant increase in CSF outflow at 45 cmH**_**2**_**O ICP.** In order to stratify the correlation of the specific drug effects at a steady-state pressure of 45 cmH_2_O, we plotted drug concentrations against CSF outflow using extrapolated data. **(A)** saline delivery, **(B)** noradrenaline (NA) at 50 μM and 500 μM (n = 5), **(C)** L-NMMA at 10 μM and 100 μM (n = 4), **(D)** U46619 at 0.1 μM and 1.0 μM (n = 5) and **(E)** U46619 at 1 mM, 25 cmH2O (n = 3). Asterisk denotes significance relative to no drug treatment (NT) (ANOVA/Dunnett’s test).

To address how ICP influences CSF absorption, outflow rates were calculated using regression analysis. The ability of a pharmacological agent to alter CSF absorption was related to the steady-state ICP, and the concentration and class of pharmacological agent delivered. Normal ICP in adult humans is approximately 10 cmH_2_O. In contrast, traumatic brain injury or intracranial hemorrhage have been found to result in ICP reaching above 40 cmH_2_O, and aggressive interventions are typically initiated when pressures exceed 30 cmH_2_O [19-21]. To mirror ICP ranges typically found in severe clinical cases, we therefore calculated CSF outflow rates using linear regression with a steady state pressure of 25 cmH_2_O and also by extrapolation of the data, at 45 cmH_2_O. At a steady state pressure of 25 cmH_2_O there was no statistically significant drug effect on CSF absorption regardless of drug concentrations or drug class (r = 0.26, *p* = 0.093) (Figure 3A). In contrast, at a steady state pressure of 45 cmH_2_O, a strong correlation was found between drug concentration and CSF absorption (r = 0.42, *p* = 0.0058) (Figure 3B). Indeed, the delivery of 100 μM L-NMMA significantly increased CSF absorption by an average of 2.29 fold over no treatment (2.29 ± 0.34 mL/min), while delivery of 1 μM U46619 resulted in an average increase of 2.44 fold in CSF absorption over no treatment (2.44 ± 0.55 mL/min) (Figure 4C and D). Similarly, at steady state pressures of 30 (see Additional file 1: Figure S1), 35 and 40 cmH_2_O (data not shown), CSF outflow rates were increased significantly relative to no treatment upon delivery of 100 μM L-NMMA and 1 μM U46619. Saline delivery did not significantly increase CSF absorption (0.88 ± 0.097 mL/min) (Figure 4A). A positive trend of increased CSF absorption with the delivery of NA was observed, however it did not reach statistical significance at any steady-state pressure (Figure 4B). Conversely, the delivery of 1 mM U46619 at 25 cmH2O significantly decreased CSF absorption by 0.71 fold (0.71 ± 0.0098 mL/min), likely due to lymphatics becoming tonic at this concentration (Figure 4E).

#### Pharmacological agents delivered intranasally change CSF outflow resistance

**Figure 5:**
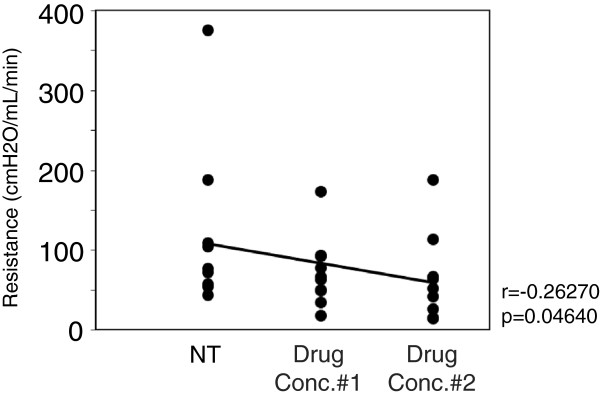
**Delivery of pharmacological agents alters CSF outflow resistance.** To address whether a correlation existed between an overall drug effect and resistance, data points from all agents under no drug treatment (NT), the delivery of the first set of agents (drug conc.#1; 50 μM NA, 10 μM L-NMMA, 0.1 μM U46619) and the delivery of the second set of agents (drug conc.#2; 500 μM NA, 100 μM L-NMMA, 1 μM U46619) were plotted against resistance to outflow, which was derived from the linear regression analysis of the raw data. The straight line linking the averages within each treatment group was calculated with the Pearson correlation test.

Given that the observed total drug effect increased overall CSF absorption (Figure 3B) we hypothesized that this would correlate with a decrease in CSF outflow resistance under steady-state pressure. Indeed, we observed that increasing drug concentrations correlated inversely with outflow resistance across all drug classes (r = -0.26, *p* = 0.046 one-way probability) (Figure 5).

## Discussion

Expansion of the ventricular space due to a deficit in CSF absorption can lead to an increase in ICP and a decrease in cerebral perfusion pressure, these scenarios being commonly seen in traumatic brain injury and hydrocephalus. With regards to the latter, the most common solution is either CSF diversion with a shunt or endoscopic third ventriculostomy (ETV), both being highly invasive with potential long-term negative consequences. From a theoretical perspective, the intranasal delivery route for pharmacological agents may provide a means to modulate CSF outflow in a non-invasive manner by targeting the lymphatic vasculature at the cribriform plate. In this report we provide proof-of-concept that supports the above hypothesis using well-characterized albeit not necessarily clinically-applicable pharmacological agents that modulate the lymphatic vasculature resulting in a change in CSF outflow.

Our data highlights a number of important considerations when modulating CSF absorption intranasally. Indeed, clear differences exist between certain classes of agents in their ability to promote CSF absorption. For example, NA appears to be less efficient in inducing CSF absorption when compared to the thromboxaneA2 analogue U46619. Consistent with this observation, *in vitro* experiments using isolated lymphatic vessels support our *in vivo* analysis [22,23], and underscore the importance of selecting and characterizing optimal concentrations and dosing of pharmacological agents that will efficiently modulate CSF absorption. Furthermore, the data suggests that the attenuation of lymphatic pumping with the delivery of L-NMMA also results in an increase in flow and a decrease in resistance. This may be related to a theoretical increase in vessel radius driven by the actions of L-NMMA. Consistent with this, an increase in vessel diameter was also observed in eNOS-/- mice as well as after NOS inhibition in control mice [24]. Additionally, in our model the efficacy of a drug appears pressure dependent; the higher the pressure, the greater the drug effect.

While our hypothesis focuses on modulating the turbinate lymphatic vasculature in order to influence CSF absorption, other pathways may play a role in augmenting CSF outflow with the most obvious being the uptake of drug into the blood vasculature. Indeed, it has been demonstrated that the intravenous delivery of catecholamines can influence lymphatic contractile activity and flow from efferent lymphatic vessels in sheep [10,25]. However, it is unclear whether the systemic circulation of pharmacological agents can ultimately influence CSF absorption via the turbinate lymphatics. Future studies to address these pathways will undoubtedly shed light on how intranasal drug delivery influences CSF absorption.

## Conclusions

We have demonstrated the utility of delivering pharmacological agents intranasally to modulate CSF absorption non-invasively. This has the potential to complement the current surgical interventions that are used to treat pathologies characterized by a deficit in CSF absorption such as hydrocephalus. Indeed, procedures to treat hydrocephalus such as ETV result in an increase in CSF drainage from the ventricles into the subarachnoid space; the augmentation of CSF absorption via the nasal lymphatics coupled with other interventions may aid in CSF clearance.

## Competing interests

The authors declare that a patent is currently under review relating to the content of the manuscript.

## Authors’ contributions

HK participated in the design of the study, data analysis and interpretation, and drafted the manuscript. SAM carried out the surgical procedures. MGJ conceived of the study and its design. All authors have read and approved the final version of the manuscript.

## Supplementary Material

Additional file 1: Figure S1 Increasing drug dose results in a significant increase in CSF outflow at 30 cmH_2_O ICP. At a constant ICP of 30 cmH_2_O, drug concentrations were plotted against CSF outflow for (A) saline delivery, (B) noradrenaline (NA) at 50 μM and 500 μM (n = 5), (C) L-NMMA at 10 μM and 100 μM (n = 4) and (D) U46619 at 0.1 μM and 1.0 μM (n = 5). Asterisk denotes significance relative to NT (ANOVA/Dunnett’s test). Click here for file
