# Supplementary figures and images for: Potential for intranasal drug delivery to alter cerebrospinal fluid outflow via the nasal turbinate lymphatics
Source: Fluids Barriers CNS. 2014 Feb 15;11:4. doi: 10.1186/2045-8118-11-4 (PMC3927830; doi:10.1186/2045-8118-11-4)

A.

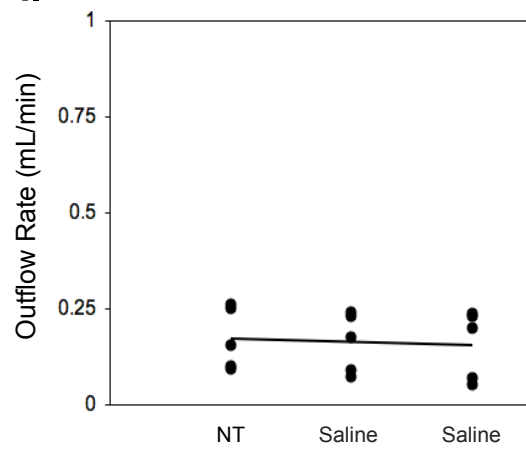

B.

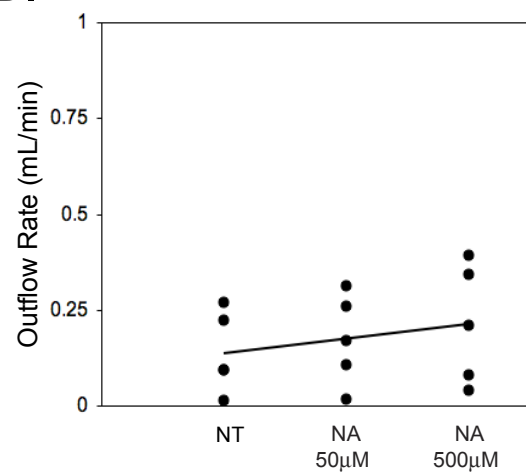

C.

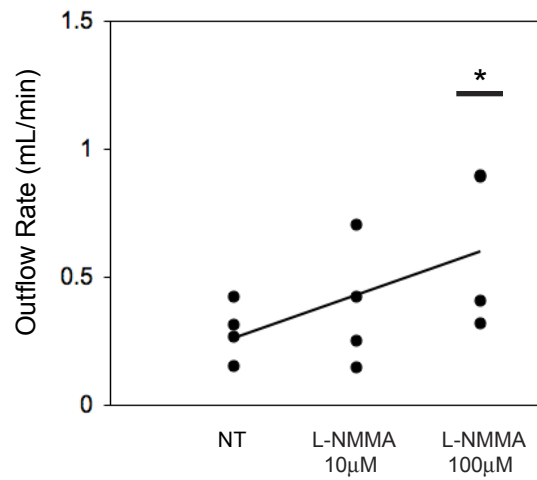

D.

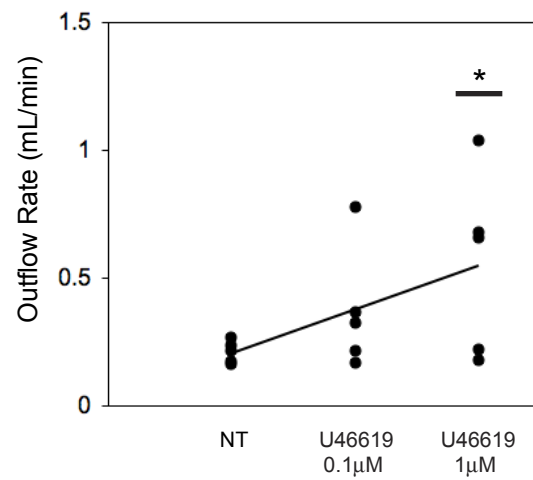

Supplement: Additional file 1: Figure S1 — Increasing drug dose results in a significant increase in CSF outflow at 30 cmH2O ICP. At a constant ICP of 30 cmH2O, drug concentrations were plotted against CSF outflow for (A) saline delivery, (B) noradrenaline (NA) at 50 μM and 500 μM (n = 5), (C) L-NMMA at 10 μM and 100 μM (n = 4) and (D) U46619 at 0.1 μM and 1.0 μM (n = 5). Asterisk denotes significance relative to NT (ANOVA/Dunnett’s test). [file 2045-8118-11-4-S1.pdf]
